# Mesenchymal stromal cells-exosomes: a promising cell-free therapeutic tool for wound healing and cutaneous regeneration

**DOI:** 10.1186/s41038-019-0178-8

**Published:** 2019-12-26

**Authors:** Peng Hu, Qinxin Yang, Qi Wang, Chenshuo Shi, Dali Wang, Ubaldo Armato, Ilaria Dal Prà, Anna Chiarini

**Affiliations:** 1 Department of Burns & Plastic Surgery, The Affiliated Hospital of ZunYi Medical University, Dalian Road 149, ZunYi City, 563000 Gui Zhou Province China; 2 Human Histology and Embryology Unit, University of Verona Medical School, Strada Le Grazie 8, 37134 Verona, Italy

**Keywords:** Cutaneous regeneration, Mesenchymal stromal cell, Exosomes, Wound healing, Microenvironment

## Abstract

Cutaneous regeneration at the wound site involves several intricate and dynamic processes which require a series of coordinated interactions implicating various cell types, growth factors, extracellular matrix (ECM), nerves, and blood vessels. Mesenchymal stromal cells (MSCs) take part in all the skin wound healing stages playing active and beneficial roles in animal models and humans. Exosomes, which are among the key products MSCs release, mimic the effects of parental MSCs. They can shuttle various effector proteins, messenger RNA (mRNA) and microRNAs (miRNAs) to modulate the activity of recipient cells, playing important roles in wound healing. Moreover, using exosomes avoids many risks associated with cell transplantation. Therefore, as a novel type of cell-free therapy, MSC-exosome -mediated administration may be safer and more efficient than whole cell. In this review, we provide a comprehensive understanding of the latest studies and observations on the role of MSC-exosome therapy in wound healing and cutaneous regeneration. In addition, we address the hypothesis of MSCs microenvironment extracellular vesicles (MSCs-MEVs) or MSCs microenvironment exosomes (MSCs-MExos) that need to take stock of and solved urgently in the related research about MSC-exosomes therapeutic applications. This review can inspire investigators to explore new research directions of MSC-exosome therapy in cutaneous repair and regeneration.

## Background

The skin is frequently damaged as a result of acute or chronic wounds such as extensive burns, trauma or ulcers of various aetiology. These injuries not only destroy the barrier function of the skin but also modify the sensory perceptions of temperature, pain and touch [1]. Besides, they constitute a painful experience for patients both physically and mentally, and also cause a huge socioeconomic burden [2]. Therefore, identifying an effective approach to accelerate cutaneous regeneration and restore the functions of the injured skin is an urgent requirment. Besides, this topic has become a significant challenge in plastic and reconstructive surgery. A novel therapy for wound healing and regeneration gaining momentum in the past few years has been the use of mesenchymal stromal cells (MSCs). MSCs reside in normal skin and play a critical role in wound healing. Therefore, the application of exogenous MSCs was proposed to promote regenerative healing of wounded skin [3]. Although great progress has been made in the application of MSCs in wound repair and cutaneous regeneration, the limitations inherent to MSCs cell therapy cannot be ignored. Therefore, the exosomes derived from MSCs (MSC-exosomes) have gained much attention as a new potential “cell-free” approach in the field of wound healing and cutaneous regeneration.

In this review, we discuss the confusing issues related to MSCs, i.e. the lack of universally accepted criteria for defining the MSCs phenotype and/or their functional properties, and discuss the current clinical trials using MSCs in wound healing therapy and their current limitations. Then we expound the roles of MSC-exosomes in cutaneous regeneration and summarize their underlying molecular mechanisms. We also clarify the current scientific problems of MSC-exosomes as the research trend of “cell-free” therapy. Finally, we bring forward the key problems which at present need to be solved and express the hope that more researchers will study this issue in depth.

## Review

### The defining and naming of MSCs

Although the biological characteristics and therapeutic potential of MSCs have been deeply studied, the behavior *in vivo* and developmental origin of MSCs have not been clarified yet. With regard to the source of MSCs, since mesenchymal stem cells exist in almost all tissues and organs, there is also a popular hypothesis that MSCs come from perivascular cells [4]. But some researchers have found that MSCs can also come from tissues without blood vessels and nerves, such as intervertebral disc [5]. Precisely defining MSCs has been a challenge, as the field is complicated by inconsistencies related to MSCs nomenclature and identification criteria. For example, bone marrow stromal cells, bone marrow stromal stem cells, MSCs, mesenchymal stem cells, and medical signal cells, all of which describe the same group of cells, the widely recognized the acronym, MSC, may be used for all of them.

Bone marrow stromal cells were first discovered and reported by Friedenstein AJ in the late 1960s. In 1988, Owen et al. defined it as bone marrow stromal stem cells on the basis of previous researchers’ work. In 1991, Caplan et al. found that there was a group of mesoderm-derived stem cells with different tissue lines in bone marrow during embryonic development, and named these cells as mesenchymal stem cells. The name has been widely recognized in the world [6]. However, some scholars believe that there are no sufficient evidences of self-renewal and multidirectional differentiation potential ability of mesenchymal stem cells *in vivo*, and their biological characteristics do not meet the standards of stem cells. It is not scientific enough to call stem cells, which may mislead patients with excessive and unrealistic expectations. Therefore, Horwitz called for the naming of these cells as “MSCs” at the International Conference on Cell Therapy [7]. The International Society for Cellular Therapy also tried to resolve challenges in confirming MSC identity by proposing three minimal criteria for defining human MSCs: (1) the cells must be plastic-adherent when maintained in standard culture conditions using tissue culture flasks; (2) ≥95% of the population must express CD105, CD73, and CD90 and ≤ 2% must not express CD45, CD34, CD14, CD11b, CD79α, or CD19, and HLA class II surface molecules; and (3) the cells must be able to differentiate into osteoblasts, adipocytes, and chondroblasts under standard in vitro differentiating conditions [8]. Furthermore, the researchers found that the conditioned medium of MSCs had biological effects equivalent to MSCs in promoting wound healing and preventing and treating organ fibrosis. It was suggested that the biological effect of MSCs mainly came from its paracrine mechanism. It was found that MSCs extracellular vesicles or exocrine bodies also had almost equivalent biological effects to MSCs. In 2010, Caplan proposed the new name of “medicinal signal cell, MSC” , because of these cells like “medicine” homing to the site of injury or disease, secreting bioactive factors, playing a treatment *in situ* [9]. In fact, no matter how precisely MSCs are defined, this does not affect the applicative prospects of these cells in treatment.

### MSCs in wound healing and cutaneous regeneration

Cutaneous wound healing is a highly organized physiological process that restores the integrity of the skin following injury. It involves the interplay between various populations of cells and is typically categorized into three reciprocally overlapping phases: inflammation, proliferation, and maturation [10, 11]. Various MSCs are also deemed to partake in this process, like the endogenous cutaneous MSCs which include dermal papilla cells (DPCs) and the dermal sheath cells (DSCs) [12–14]. And the perivascular pericytes and MSCs residing in the adipose tissue may act as MSCs *in vivo *[15–17]. However, in most trauma cases, such as deep burns and chronic ulcers, there are not enough endogenous cutaneous MSCs to partake in wound self-repair because of the missing dermal tissue. Therefore, exogenous MSCs have been applied to wounds to exploit their physiological therapeutic actions in wound healing. Regardless of the caveats in their identity or source, MSCs were reported to exert beneficial effects on both wound healing and scarring. Since MSCs are endowed with a wide differentiation potential, they are attractive treatment options in regenerative medicine and, over the past decade, have rapidly emerged as a cell therapeutic tool for advancing wound healing.

Most of the evidence related to MSCs activity in wound healing comes from bone marrow-derived MSCs (BMSCs) and other tissue of origin MSCs used in animal models, with only a small number of published clinical studies. In addition, these clinical studies are based on the transplantation of autologous BMSCs [18–21]. BMSCs are collected via bone marrow aspiration, which is a safe but painful and invasive procedure, sometimes associated with complications such as infection and hemorrhage [22]. Additionally, bone marrow is a limited resource; there occurs an age-dependent reduction in bone marrow cells numbers [23]. Therefore, MSCs of several different sources have been used as alternative choices, including adipose-derived stromal cells (ADSCs), dermal MSCs, and MSCs from amniotic fluid and umbilical cord. ADSCs and dermal MSCs are abundantly available in fat and skin tissues, can be harvested with minimally invasive procedures, and their use is devoid of ethical controversies making them good alternatives to BMSCs. ADSCs and dermal MSCs have similar biological characteristics, immunogenicity, and potential to differentiate to BMSCs [24–29] . In clinical trials currently under evaluation [30], ADSCs have demonstrated a therapeutic potential in burn wounds and ulcers. Likewise, dermal MSCs have shown beneficial effects on wound healing in clinical trials [31, 32]. Although, there are no clinical trials related to human amniotic membrane-derived MSCs (hAM-dMSCs) and human umbilical cord-derived MSCs (hUC-dMSCs) in wound healing have appeared currently. These promising clinical studies indicate that MSC-based therapies are safe and potentially efficacious, with no indication that any particular MSC tissue origin has an advantage for wound healing over the others [12].

### Current limitations of MSCs application in wound healing

Despite progression in MSC-based therapies, many challenges to overcome before MSCs can be used for effectively for wound healing treatment. The drawbacks in the clinical application of MSCs have emerged gradually: Firstly, there is considerable heterogeneity in the delivery protocols, wound models, and MSCs populations among published studies which makes it hard to determine the impact of timing of delivery, number of cells delivered, and site of delivery on MSCs engraftment upshots [33]. Secondly, there is no evidence that MSCs differentiate into phenotypes typical of resident cutaneous cells during skin wound healing [34]. Increasing evidences suggest that MSCs secrete bioactive factors through endocrine and paracrine pathways to target tissues and cells, thereby reducing wound inflammation and promoting tissue repair [33]. So the criteria to assess the need for direct MSCs transplantation onto wounds are to be determined. Finally and importantly, current challenges with the use of MSCs concern the lack of universally accepted criteria for defining the MSCs phenotype or their functional properties, and further clinical trials are needed to demonstrate the potential therapeutic benefits of MSCs in vast cohorts of patients [33].

### Exosomes derived from MSCs (MSC-exosomes)

Exosomes are cell-derived vesicles that are present in many and perhaps all eukaryotic fluids, including blood, urine, and growth media of cell cultures [35]. The reported diameter of exosomes is between 30nm and 100 nm, which is larger than low-density lipoproteins (LDL) but much smaller than, for example, other vesicles or red blood cells. Exosomes are either released from the cell when multivesicular bodies fuse with the plasma membrane or released directly from the plasma membrane [36]. Reportedly, the contents within exosomes are manifold including cytokines, proteins, lipids, mRNAs, miRNAs, noncoding RNAs (ncRNAs), ribosomal RNAs (rRNAs), cytokines and chemokines [37]. Accumulating lines of evidence shown that exosomes have specialized functions and play keys role in processes such as coagulation, intercellular signaling, and waste products management [38]. Consequently, the interest in the clinical applications of exosomes is growing. Their functions include immune regulation, vascular regeneration promoting, mediation of cell proliferation, differentiation, migration and apoptosis, preserving the body physiological condition, and partaking in disease processes [39].

MSC-derived exosomes (MSC-exosomes) exhibit the characteristics of resource cells, which can promote cell self-repair and tissue regeneration, restore tissue homeostasis and accelerate wound repair in injury areas [40]. In recent years, some studies have suggested that MSCs have a strong ability to produce exosomes [41]. Most researchers believe that MSC-exosomes are the main effective paracrine component of MSCs and play biological effect almost equivalent to those of whole MSCs. In comparison with MSCs, MSC-exosomes have the following advantages: First, MSC-exosomes exert intense biological effects because they directly fuse with target cells. Second, MSC-exosomes can be stored and transported at − 70 °C for a long time since their effective components are protected by the exosome’s plasma membrane, which is not easy to be destroyed. Third, the concentration, dose, route and time of use are easy to control. Last but not least, there is no risk of immune rejection and tumorigenesis caused by cell transplantation therapy [42].

### MSC-exosomes in wound healing and cutaneous regeneration

The typical skin regeneration process can be summarized as three overlapping stages: inflammation phase, proliferation phase (cell proliferation and reepithelization) and remodeling phase [10–12]. Currently, all the researches on MSC-exosomes in wound healing and cutaneous regeneration are also focussed on the role of MSC-exosomes in the above three stages.

#### Mechanism of MSC-exosomes behavior on inflammation phase

Inflammation is a body self-defense mechanism in response to harmful stimuli, and an acute and well-regulated inflammatory response is beneficial for normal wound healing [43]. By contrast, a chronic and dysregulated inflammatory response may delay wound healing and promote fibrosis, excessive scar formation or inhibition of re-epithelialization [44]. Macrophages are prominent inflammatory cells which play an important role in the cutaneous regeneration process. Recent evidence has suggested that macrophages influence each stage of cutaneous regeneration and present a proinflammatory M1 phenotype and an anti-inflammatory M2 phenotype. Macrophage dysfunction may promote excessive inflammation or fibrosis [45]. MSC-derived extracellular vesicles, including MSC-exosomes, promote the significant switching of recipient’s macrophages toward the anti-inflammatory M2 phenotype [46]. In addition, MSC-exosomes can regulate the activation, differentiation, and proliferation of B lymphocytes and can also suppress T-lymphocyte proliferation. MSC-exosomes can convert activated T lymphocytes into the T-regulatory phenotype, thereby exerting immunosuppressive effects [47, 48]. Furthermore, the regulation of inflammatory factors plays an important role in skin tissue regeneration, and excessive production of cytokines may lead to tissue injury [49]. Exosomes derived from different kinds of MSCs can mitigate the inflammatory response caused by multiple stimuli through the down-regulation of proinflammatory enzymes, like inducible nitric oxide synthase (iNOS) and cyclooxygenase (COX)-2, and of cytokines and chemokines, like tumor necrosis factor (TNF)-α, interleukin (IL)-1β and monocyte chemoattractant protein (MCP)-1. Moreover, in many disease models, MSCs-exosomes can drive the up-regulation of an anti-inflammatory cytokine, i.e. IL-10, which reportedly plays a critical role in the control of cutaneous wound inflammation and scar formation [50–52]. Various studies have revealed that MSC-exosomes exert immunomodulatory effects through specific miRNAs. By analyzing miRNA expression profiles, they showed that hUC-dMSCs exhibit the highest content of three miRNAs (miRNA- 21, miRNA-146a and miRNA-181c) specifically related to the regulation of immune response and inflammation [53, 54]. Furthermore, other studies showed that hUC-dMSCs-exosomes carrying miRNA-181c attenuated the burn-induced very intense inflammation by down-regulating the Toll-like receptor 4 (TLR4) signaling pathway [51]. Anti-inflammatory miRNAs (miRNA-124a, miRNA-125b) detected within exosomes have been implicated in the post-transcriptional silencing of chemokines and cytokines (e.g., TNF-α and MCP-1) which instead contribute to the persistence of inflammatory cellular infiltrations in wound healing [55, 56]. Overall, the specific molecular mechanisms through which MSC-exosomes inhibit inflammation in the setting of wound healing and cutaneous regeneration also need to be clarified by further studies.

#### Mechanism of MSC-exosomes behavior on proliferation phase

In the proliferation phase, neoangiogenesis, collagen deposition, granulation tissue formation, re-epithelialization, and wound contraction concur [57]. The formation of neoangiogenesis is a crucial step in various physiological and pathological processes, including wound healing and tissue repair [10, 58, 59]. MSC-exosomes are enriched in various angiogenesis-related proteins and RNAs, including miRNAs that could activate multiple signaling pathways in endothelial cells. The same exosomes can induce the expression of numerous trophic factors [60]. In addition, Li et al.revealed that transplanted human umbilical cord blood endothelial progenitor cells-derived exosomes (EPC-exosomes) could upregulate the expression of angiogenesis-related molecules, including vascular endothelial growth factor (VEGF) - A, vascular endothelial growth factor receptor (VEGFR)-2, fibroblast growth factor (FGF)- 1, E-selectin, angiopoietin-1, Chemokine (C-X-C motif) ligand 16, IL- 8, and endothelial nitric oxide synthase (eNOS), in vascular endothelial cells [61]. Furthermore, the mRNA levels of matrix metalloproteinase (MMP)-9 were remarkably decreased in endothelial cells stimulated with EPC-exosomes [61]. The study has found that significantly higher expression of MMP-9 was associated with poor wound healing [62]. Thus, the proangiogenesis effects of EPC-exosomes may be partially attributed to their inhibition of MMP-9. However, it is still necessary to reveal their further mechanisms about angiogenesis in wound repair.

Cell proliferation and skin re-epithelization are crucial for cutaneous regeneration. Skin fibroblasts play a relevant role in skin tissue repair and regeneration: they participate in wound contraction, extracellular matrix deposition, tissue remodeling, and so on [63]. MSC-exosomes can be internalized and hence transport their contents, such as proteins and RNAs, into the receiving cells to regulate their proliferation and migration. It has been proven that MSC-exosomes regulate the proliferation and migration of fibroblasts by modulating the expression of growth factors and their related genes [64–66] thus taking part to the formation of granulation tissue and to the synthesis of collagen which provides structural support for wound repair [67, 68]. Some scholars treated fibroblasts extracted from chronic diabetic ulcer wounds with exosomes derived from BMSCs. The results showed that exosomes could promote the proliferation and migration of fibroblasts in a dose-dependent manner. Human fibrocyte-derived exosomes contain proteins and miRNAs with diverse biological activities, and these exosomes accelerated wound healing by inducing the migration and proliferation of skin cells in the diabetic rat model [55].

In addition, the study found that exosomes derived from hUC-dMSCs promoted the proliferation of skin cells (dermal fibroblasts, epidermal keratinocytes) in a dose-dependent manner. In in vivo experiments, the researchers used local multi-point injection to evaluate the effect of exosomes by injecting exosomes around deep II degree burn wounds models. The results showed that exosomes derived from hUC-dMSCs accelerated wound healing, promoted re-epithelialization, and increased the expression of cytokeratin-19 (CK19), proliferating cell nuclear antigen (PCNA) and collagen I [69, 70]. Moreover, transplanting human amniotic epithelial cell-derived exosomes (hAEC-exosomes) to wound sites accelerated wound closure and re-epithelization [71].

#### Mechanism of MSC-exosomes behavior on remodeling phase

Extracellular matrix (ECM) mainly constituents of four kinds of substances, namely collagen, non-collagen (fibronectin, laminin), elastin, proteoglycans, and aminoglycans. The key to ECM reconstruction is the synthesis and degradation of collagen. Insufficient or excessive ECM formation can cause the wound surface not to heal or scar formation. MSC-exosomes have been shown to regulate ECM re-synthesis in addition to participating in the above cellular effects. Reportedly, exosomes derived from hUC-dMSCs can promote the de novo synthesis of type I collagen and elastin [65], and exosomes from the inducing process which human induced pluripotent stem cells (hiPSCs) had differentiated to dermal MSCs (dMSCs) can promote the synthesis of type I collagen, type III collagen, and elastin proteins, and enhance the expression levels of type I collagen, type III collagen, and elastin mRNAs [72]. These findings prove that MSC-exosomes can promote the regeneration of ECM and thus advance wound healing. The exosomes derived from ADSCs (ADSCs-exosomes) can also regulate collagen synthesis at different stages of wound healing, accelerate wound healing through an early stage increase in the production of type I and type III collagen, and inhibit collagen synthesis in a late stage, thereby reducing scar formation [73]. In a skin-defect mouse model, exosomes derived from hUC-dMSCs did prevent scar formation by inhibiting the differentiation of fibroblasts into myofibroblasts [74]. Moreover, the research reported that hAEC-exosomes exerted beneficial effects in a rat model of scarless wound healing. High concentration of hAEC-exosomes partly reduced ECM deposition by stimulating the expression of MMP-1 [71]. In addition, Wang et al. posited that ADSCs-exosomes can inhibit the formation of type III/type I collagen by directly acting on fibroblasts [75]. In a study applying traceable ADSCs-exosomes to repair skin defects in mice, the exosomes were recruited around the skin wounds to perform their functions and to increase the rate of wound healing. Histological analysis showed that exosomes could promote collagen synthesis in the early stage of wound healing, and inhibit collagen synthesis in the late stage to inhibit scar tissue formation [64] .All these studies indicate that MSC-exosomes play a key role in ECM remodeling, which is also the possible mechanism of reducing scar formation.

### Hypothesis of MSCs-MEV or MSCs-MExos and future perspectives

Over the past decades, a deep-going research has taken place concerning MSCs and MSC-exosomes roles in the field of wound repair and cutaneous regeneration. MSC-exosomes based therapy is emerging as a promising technique able to promote wound healing and minimize scarring. Accordingly, being a cell-free alternative therapy, MSC-exosomes enjoy many advantages, being easy to be prepared, stored and transported, easy to be dosed, and easy to be administered at the time of choice. They also appear to have a high therapeutic efficiency, and to carry no risk of immune rejection and tumorigenesis. Thus, MSC-exosomes are endowed with a remarkable potential for cutaneous regeneration and could effectively replace whole MSCs-based therapy. The molecular mechanisms through which the MSC-exosomes bring into effect cutaneous regeneration will be fully brought to light by further studies looking into their specific contents and functions. At present, most of the mechanisms discussed were studied in rodents, but animal physiology cannot always be extrapolated to humans. So further clinical trials using exosomes of human origin are needed to definitively demonstrate the skin regenerative therapeutic potential of MSCs in a large number of patients. However, there are still key scientific issues that need to be resolved before.

Although MSCs derived from different tissues do not have the same differentiation potency, their physical and biological functions show a high level of consistency [76, 77]. So it might not be considered as an obstacle that which MSCs should be chosen to produce exosomes. However, MSCs transplantation has different biological effects in different stages of wound healing or in different diseases. In fact, the fate and paracrine effects of MSCs are closely related to the microenvironment of their transplantation site [78, 79]. For instance, studies have shown that transplantation of MSCs into organs and tissues can up-regulate the expression of VEGF and accelerate the regeneration and healing of injured tissues [80]; But in some studies concerning tumors, researchers have found that MSCs can down-regulate the expression of VEGF and inhibit the angiogenesis supporting the growth of tumors [81, 82]. Our studies also corroborate that MSCs derived from different sources can promote fibroblast proliferation and migration through the paracrine effect, and up-regulate the expression of transforming growth factor (TGF) -β1, collagen I and angiogenesis factors in fibroblasts and wound tissues to advance the formation of granulation tissue and accelerate wound re-epithelialization. But when MSCs were transplanted after wound re-epithelialization, the biological effects were changed by the paracrine effect in *in vivo* and *in vitro* studies (e.g., the expression of fibrosis-related factors such as TGF-β1, α-smooth muscle actin (α-SMA) and collagen I was down-regulated, and the expression of anti-fibrosis factors such as TGF-β3 and Decorin was up-regulated to hinder the formation of the scar.

**Fig. 1:**
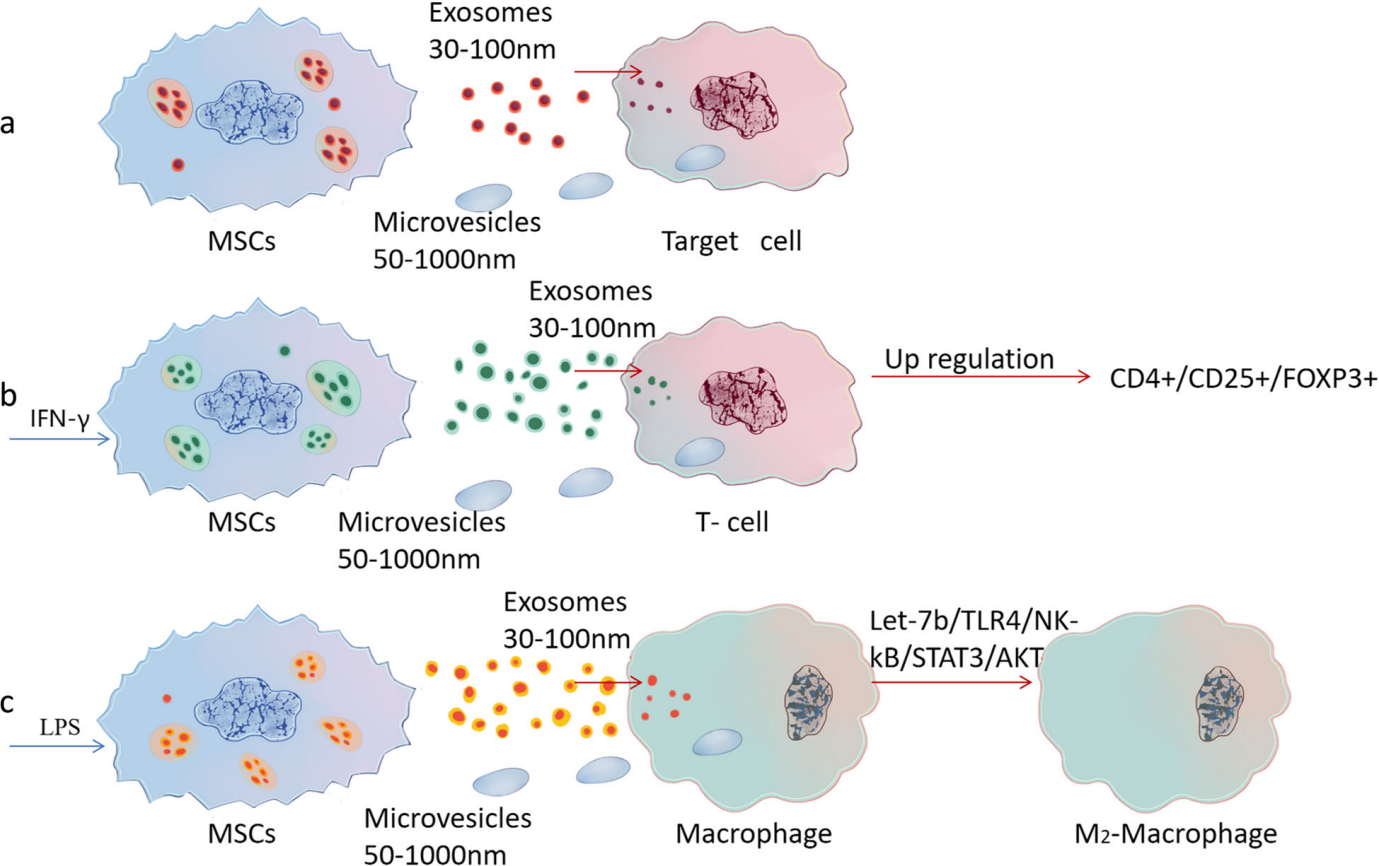
Under the stimulating human umbilical cord-derived mesenchymal stromal cells (hUC-dMSCs) with different factors, the quantities and components of exosomes had changed. Then, the biological characteristics of target cells also have changed after received the exosomes. (**a**) In a normal situation, target cells receive microvesicles and exosomes released from MSCs. (**b**) Stimulating hUC-dMSCs with interferon (IFN)- g, there were in a simulated inflammatory microenvironment that can significantly increase the release of MSCs-exosomes. Then, it can enhance the immunomodulatory activity of hUC-discs and increase the proportion of CD4+, CD25+ and Foxp3+T cells in the entire regulatory T cells. (**c**) Stimulating hUC-dMSCs with lipopolysaccharide (LPS) can secrete more exosomes than untreated ones, and the exosome derived from them are rich in miRNA let-7b, which can induce macrophages to exhibit the anti-inflammatory M2 phenotype by the specific signal pathway

**Fig. 2:**
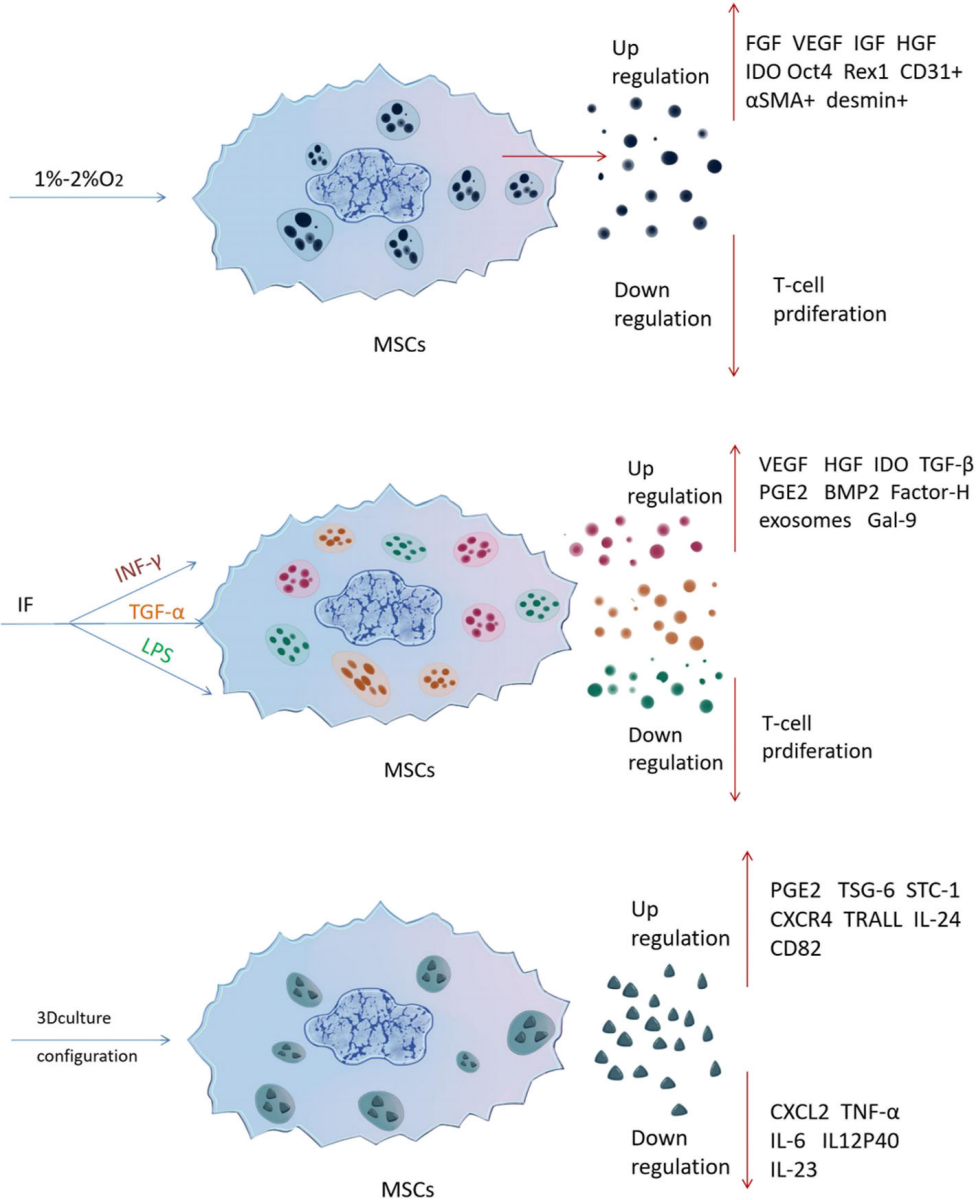
With the changing of culture environment, the components of mesenchymal stromal cell - extracellular vesicles (MSCs-EVs) or MSCs-exosomes also have changed. *IFN* interferon, *IL* interleukin, *TGF* transforming growth factor, *TNF* , tumor necrosis factor, *VEGF* vascular endothelial growth factor, *α-SMA* α-smooth muscle actin

Recent research also has shown that stimulating hUC-dMSCs with interferon (IFN) -γ if there were in a simulated inflammatory microenvironment can significantly increase the release of MSCs-exosomes. IFN-γ can enhance the immune regulatory activity of hUC-dMSCs acting like a “license” factor and increase the proportion of CD4^+^, CD25^+^ and Foxp3^+^ T cells among the whole Regulatory T cells (Tregs) [83]. Lipopolysaccharide (LPS) pretreated hUC-dMSCs secrete more proteins than untreated ones, and the exosome derived from LPS-pretreated hUC-dMSCs are rich in miRNA let-7b, which can induce macrophages to take on the anti-inflammatory M2 phenotype [53]. (Fig. 1). In addition, the research showed that 1–2% hypoxia environment, media with TNF-α, and three-dimensional culture system all had been found significantly change the components of MSCs paracrine factors* in vitro *culture [84]. (Fig. 2) The release and components of MSC-exosomes can be modulated by changing the cell culture conditions, that is the microenvironment in which the cells live.

However, all the current studies on the mechanisms of MSC-exosomes activities are based on the MSC-exosomes isolated from the standard culture medium *in vitro*. This obviously contradicts the important scientific argument that the paracrine effect of MSCs is closely related to their microenvironment. In other words, MSC-exosomes secreted in different microenvironments have different components which exert dissimilar biological effects. This important scientific issue has been ignored by almost all researchers of studying MSCs-EVs or MSC-exosomes.

## Conclusions

Wound healing and cutaneous regeneration is a complicated and dynamic process, and the same is true of other diseases. If we only isolate MSC-exosomes from conditioned medium supernatants under standard laboratory culture conditions, then use them as a cell-free therapy for different diseases or different pathological stages of the same disease, it will be obviously difficult to “accurately” achieve an effective cell therapy based on MSCs.

Even if, we can not fully elucidate its essence and mechanism of inducing MSCs paracrine effect, and it is difficult or even impossible to simulate the microenvironment completely at present. However, we can imitate it in the vitro culture systems which are based on the known environmental factors, such as inflammatory factors in wound inflammation stage, and hypoxia factors in ischemic anoxic wounds. There is no doubt, the complexity of wound microenvironment should not be the excuse of ignoring the microenvironmental factors in studying MSCs-EVs or MSCs-Exos.

Therefore, we propose the hypothesis of MSCs-MEV or MSCs-MExos which takes stock of the conditions in which the exosomes were produced as related to their therapeutic applications. We also hope that more scholars will pay a greater attention to this scientific problem, which is to find more and more suitable methods simulating the damaged site microenvironment of diverse diseases or of different pathological stages of the same illness. If these key problems can be solved, MSCs will have a significant impact on wound healing and cutaneous regeneration, besides other diseases, as a cell-free therapy.

## Data Availability

Not applicable.

## Abbreviations

ADSCs: Adipose-derived stromal cells
ADSCs-exosomes: Exosomes derived from ADSCs
BMSCs: Bone marrow-derived MSCs
CK19: Cytokeratin-19
COX: Cyclooxygenase
dMSCs: Dermal mesenchymal stromal cells
DPCs: Dermal papilla cells
DSCs: Dermal sheath cells
ECM: Extracellular matrix
eNOS: Endothelial nitric oxide synthase
EPC-exosomes: Endothelial progenitor cells-derived exosomes
FGF: Fibroblast growth factor
hAEC-exosomes: Human amniotic epithelial cell-derived exosomes
hAM-dMSCs: Human amniotic membrane-derived mesenchymal stromal cells
hUC-dMSCs: Human umbilical cord-derived mesenchymal stromal cells
hiPSC: Human induced pluripotent stem cells
IFN: Interferon
IL: Interleukin
iNOS: Inducible nitric oxide synthase
LDL: Low-density lipoproteins
LPS: Lipopolysaccharide
MCP: Monocyte chemoattractant protein
miRNAs: MicroRNAs
MMP: Matrix metalloproteinase
mRNA: Messenger RNA
MSC-exosomes: Exosomes derived from MSCs
MSCs: Mesenchymal stromal cells
MSCs-MEVs: MSCs microenvironment extracellular vesicles
MSCs-MExos: MSCs microenvironment exosomes
ncRNAs: noncoding RNAs
PCNA: Proliferating cell nuclear antigen
TGF: Transforming growth factor
TNF: Tumor necrosis factor
Tregs: T cells
VEGF: Vascular endothelial growth factor
VEGFR: Vascular endothelial growth factor receptor
X-C motif: Chemokine
α-SMA: α-smooth muscle actin
