# Corrigendum to: ‘Mesenchymal stromal cells-exosomes: a promising cell-free therapeutic tool for wound healing and cutaneous regeneration’

**DOI:** 10.1093/burnst/tkaa007

**Published:** 2020-01-23

**Authors:** Peng Hu, Qinxin Yang, Qi Wang, Chenshuo Shi, Dali Wang, Ubaldo Armato, Ilaria Dal Prà, Anna Chiarini

**Affiliations:** 1 Department of Burns & Plastic Surgery, The Affiliated Hospital of ZunYi Medical University, Dalian Road 149, ZunYi City 563000, Gui Zhou Province, China; 2 Human Histology and Embryology Unit, University of Verona Medical School, Strada Le Grazie 8, 37134 Verona, Italy

The published version of this Review neglected to state that this work was supported by the INVITE Project—European Union's Horizon 2020, an innovation programme under the Marie Sklodowska-Curie grant agreement No. 754345.

## Supplementary Material

Supplementary_Figure_1_tkaa007

