# Supplementary material for: Corrigendum to: ‘Mesenchymal stromal cells-exosomes: a promising cell-free therapeutic tool for wound healing and cutaneous regeneration’
Source: Burns Trauma. 2020 Jan 23;8:tkaa007. doi: 10.1093/burnst/tkaa007 (PMC7175761; doi:10.1093/burnst/tkaa007)

Supplementary Figure 1. Morphology of keratinocytes in response to Pirfenidone (PFD) treatment. Shown are representative images of normal (top row) and keloid (bottom row) keratinocytes, without PFD treatment (left images) or with 24 hrs treatment with 200 μg/ml PFD (center images) or 400 μg/ml PFD (right images). Note that normal keratinocytes form large colonies characterized by tightly adhered cells, without or with PFD treatment. In contrast, untreated keloid keratinocytes display colonies characterized by loosely associated cells. Further, untreated keloid keratinocytes appear more refractory in phase-contrast microscopy, indicating cells that are rounded up and less tightly adhered to the tissue culture dish. PFD-treated keloid keratinocytes are less refractory and form tighter colonies than untreated keloid cells, similar to normal keratinocytes.

24 hr

NTX | 200 µg/mL PFD | 400 µg/mL PFD

Normal Keratinocytes

Keloid Keratiniocytes

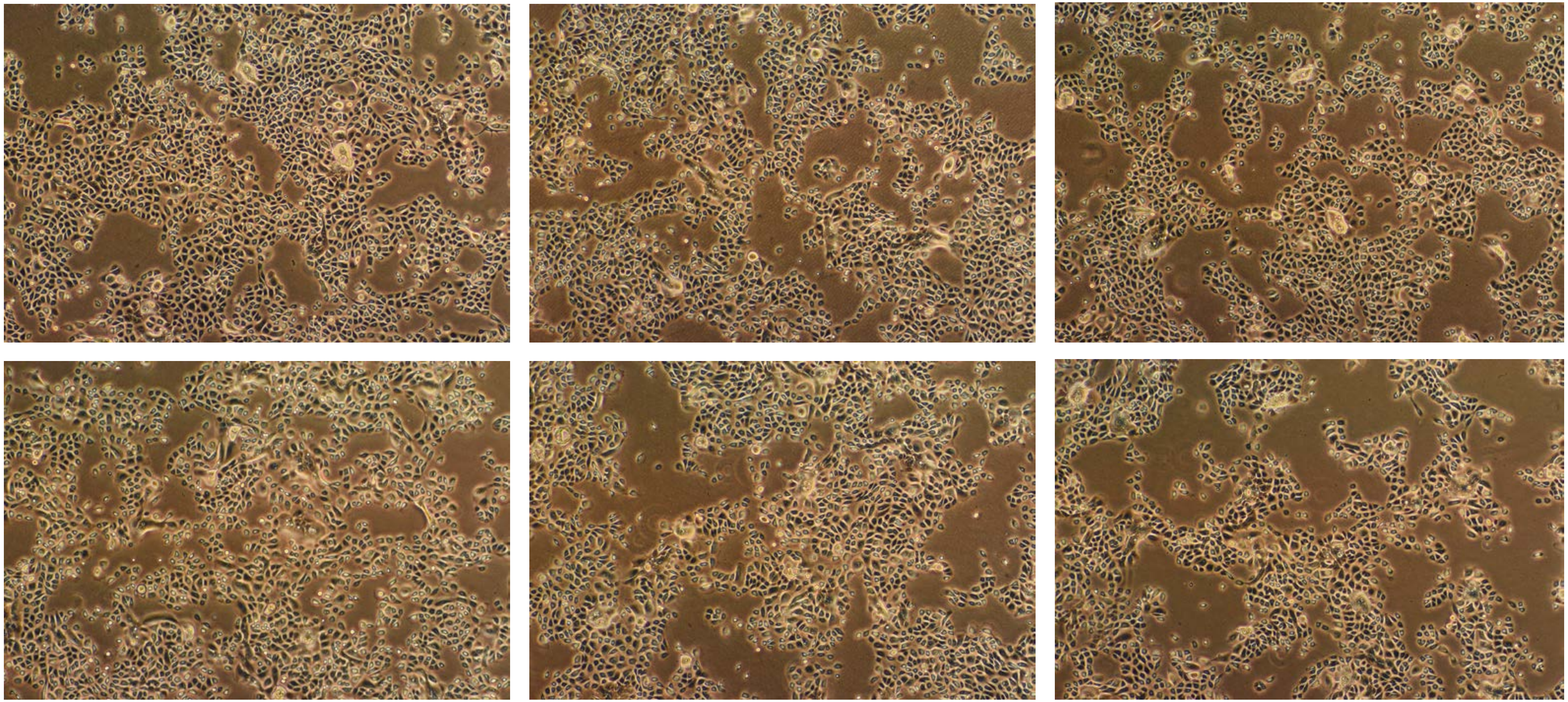

Supplement: Supplementary_Figure_1_tkaa007 [file supplementary_figure_1_tkaa007.pdf]
